# Effect of chromium on the corrosion behaviour of low Cr-bearing alloy steel under an extremely high flow rate

**DOI:** 10.1039/d0ra04334f

**Published:** 2020-09-24

**Authors:** Bei Wang, Yun Wang, Qingping Li, Huixin Li, Lei Zhang, Minxu Lu

**Affiliations:** University of Science and Technology Beijing Beijing 100083 China zhanglei@ustb.edu.cn; CNOOC Research Institute Beijing 100028 China

## Abstract

The effect of chromium on the corrosion behavior of low Cr-bearing alloy steel in a wet gas pipeline with a high flow rate was studied using a rotating cylinder electrode (RCE) and self-built wet gas flow loop device. The results show that the addition of chromium in the steel can increase the flow accelerated corrosion (FAC) resistance of steel effectively. It was hard for pure FeCO_3_ to deposit onto the carbon steel surface to form an intact corrosion film when the flow rate or wall shear stress was high. However, a mixture of Cr(OH)_3_ and FeCO_3_ can still be deposited onto the 3Cr steel surface and form an intact and protective corrosion film even under conditions with a 212 Pa wall shear stress in the wet gas pipeline.

## Abstract 2

The effect of chromium on the corrosion behavior of low Cr-bearing alloy steel in a wet gas pipeline with a high flow rate was studied using a rotating cylinder electrode (RCE) and self-built wet gas flow loop device.

## Graphical abstract

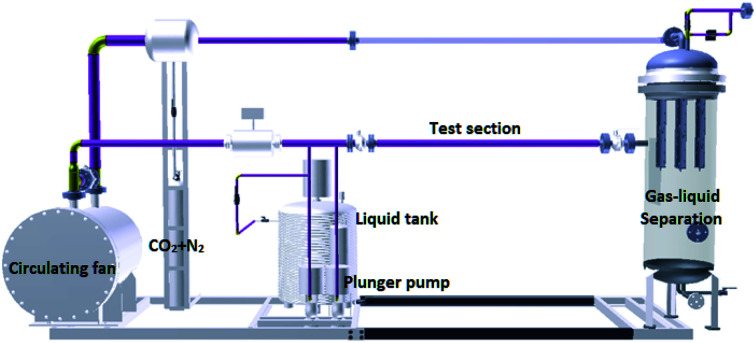

## Introduction

Carbon steel is the most widely used material in the natural gas industry. However, with the development of natural gas extraction, injection, and transportation technology, the natural gas flow rate in some areas has reached a high level. Under the combined action of corrosive substances such as CO_2_, H_2_S, and Cl^−^, carbon steel is faced with severe flow accelerated corrosion (FAC) problems.^1–4^ FAC is a deterioration action due to the effect of fluid flow that leads to the destruction or thinning of pipelines.^5,6^ FAC depends on hydrodynamic parameters of the fluid flow, such as flow rate and wall shear stress.^7^ High flow rate and wall shear stress lead to efficient mixing, which prevents the formation of protective films on the surface of the pipeline and increases the corrosion rate as a result.^8^ FAC has caused a large number of failures in piping and equipment in all types of fossil, industrial steam, and nuclear power plants, and it is a predominant mode of failure of pipelines.^9^

As a countermeasure, replacement of the carbon steel with low Cr-bearing alloy steel, which boasts an excellent performance to price ratio and improved CO_2_ corrosion resistance, has been suggested because trace amounts of Cr element can effectively prevent FAC.^10–12^ Jiang *et al.*^13,14^ reported that the addition of Cr generates amorphous products in a rust layer, and Cr is suitable for improving the corrosion resistance of low alloy steel to flow-accelerated corrosion caused by the inhibition of a cathodic reaction in an O_2_-containing environment. A large number of studies have shown that the amorphous product film will form on the surface of low Cr steel, with fewer pores and better protection.^15–17^ However, there is still a lack of research on the FAC of Cr-bearing alloy steel in the CO_2_-containing environment. Wang *et al.*^18^ found that pitting corrosion of 1Cr and 3Cr steel may occur under the flow conditions with sand. Stack *et al.*^19^ found that increases in flow velocity resulted in higher current densities for the anodic reaction. Flow rate has a significant effect on the precipitation kinetics, morphology, and mechanical properties of a FeCO_3_ scale on carbon steel.^20–22^ However, few investigations have been conducted on the effect of flow on the corrosion product performance of low Cr-bearing alloy steel in the CO_2_-containing environment.

Presently, most research on FAC is generally carried out under the liquid phase and focused on the nuclear power plant pipeline.^23–28^ However, FAC in the natural gas pipeline usually occurs in the wet gas medium, and its corrosion environment is very different from that in the liquid phase.^29–31^ Traditional laboratory liquid phase corrosion simulation methods may not be able to obtain the actual corrosion resistance and corrosion resistance mechanism of the material in the wet gas medium.

Therefore, in this study, the corrosion resistance and corrosion mechanism of carbon steel and 3Cr steel under high flow rate and high wall shear stress conditions were studied by combining the traditional experimental method and the self-built wet gas flow loop. The results of this research have essential engineering significance for the extension of 3Cr steel and the control of FAC in the natural gas pipeline.

## Experiment

### Material and solution

The materials used here were carbon steel and 3Cr steel with chemical compositions (wt%) listed in Table 1. The specimens were ground to 800 grit and then cleaned with distilled water and alcohol. The composition of the RCE test and flow loop test solutions are shown in Tables 2 and 3, respectively. Before the test, the solutions were first deaerated by pure CO_2_.

**Table 1:** Chemical composition of carbon steel and 3Cr steel (wt%)

Materials	C	Cr	Mo	Si	Mn	Nb	Fe
Carbon steel	0.05	—	0.16	0.17	1.51	0.04	Bal.
3Cr	0.07	2.96	0.15	0.20	0.55	0.03	Bal.

**Table 2:** Composition of the RCE test solution (mg L^−1^)

Composition	NaCl	MgCl_2_·6H_2_O	CaCl_2_	KCl	NaHCO_3_	Na_2_SO_4_
Concentration	25 318.99	1920.03	2747.25	643.75	519.15	196.73

**Table 3:** Composition of the flow loop test solution (mg L^−1^)

Composition	NaCl	CaCl_2_
Concentration	300	350

### RCE test

**Fig. 1:**
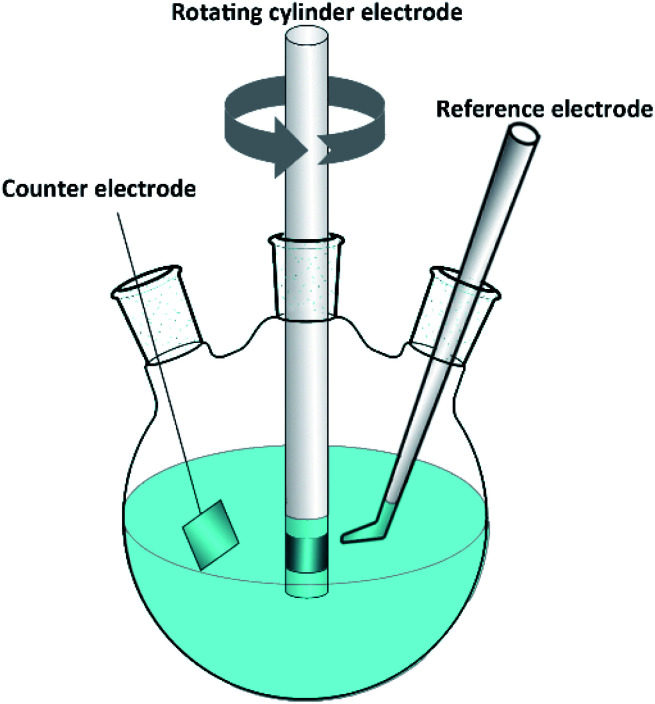
Schematic of the rotating cylinder electrode tests.

The RCE is an ideal tool for studying turbulent flow. Higher turbulent velocities are easily accessible at higher rotation rates. Fig. 1 shows a schematic diagram of the RCE. All electrochemical measurements were performed in a glass cell with a traditional three-electrode system using a Gamry Reference600+ electrochemical workstation. The volume of the solution used was 1 L. The working electrodes were carbon steel and 3Cr steel rotating cylinders, 12 mm diameter, 8 mm long, and 3 cm^2^ electrode surface area. A platinum sheet was used as a counter electrode, and a saturated calomel electrode (SCE) was used as a reference electrode.

The electrochemical impedance spectroscopy (EIS) measurements were performed using the AC signals of the 10 mV peak-to-peak amplitude at the OCP in the frequency range of 100 kHz to 5 mHz.

### Flow loop test

**Fig. 2:**
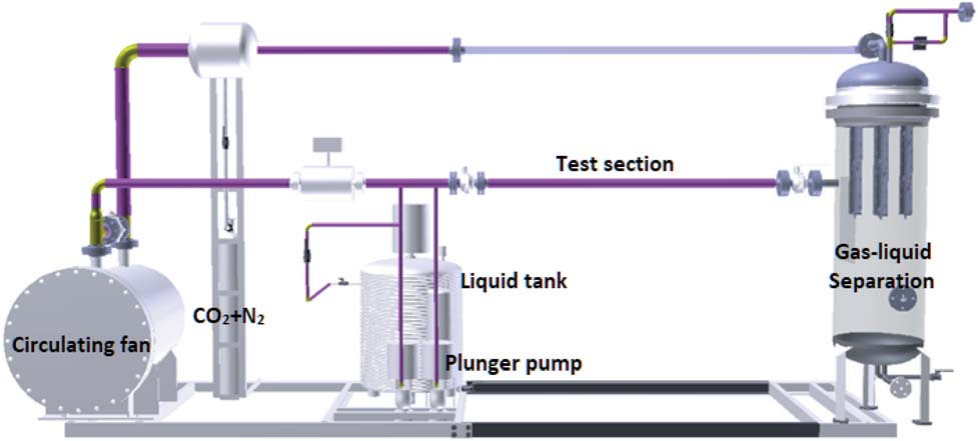
Schematic of the flow loop tests.

A schematic diagram of the flow loop is shown in Fig. 2. The flow loop is a 25/50 mm diameter, high-pressure system. The entire flow loop is manufactured from 316L stainless steel. The flow loop was driven by a circulating fan. The solution was dosed into the flow loop by a continuous dosing device and mixed with the high flow rate CO_2_ gas. The coupon was a disc with a diameter of 16 mm and a thickness of 3 mm. Three coupons were installed in the test section. A cooling casing is installed outside the test section to control the temperature of the system. Downstream of this test section, a gas–liquid separator was used to separate the liquids and gas. After gas–liquid separation, the gas phase continues to circulate in the loop, and the liquid phase is discharged to the outside. The parameters of the flow loop tests are shown in Table 4.

**Table 4:** Parameters of the flow loop tests

No.	*T*/°C	*P*/MPa	*P*_CO_2__/MPa	Water cut/%	Gas velocity/ms^−1^	*t*/h	Shear stress/Pa
1	60	0.42	0.42	0.000100	21.0	48	16.2
2	60	0.43	0.43	0.000235	31.4	48	29.3
3	60	0.43	0.43	0.000235	31.4	24	29.3
4	60	1.00	1.00	0.000100	17.6	48	19.9
5	60	0.52	0.52	0.000615	17.6	48	22.9
6	60	0.52	0.52	0.001000	40.0	72	56.0
7	60	0.52	0.52	0.001000	40.0	24	56.0
8	60	0.52	0.52	0.000615	42.0	48	84.1
9	60	1.00	0.52	0.000615	40.0	48	111
10	60	1.70	0.52	0.000615	31.4	48	212

The corrosion rate was measured using the weight-loss method. The morphology and energy dispersive X-ray spectroscopy (EDS) analysis of the corrosion scale were investigated using a JSM-6510A SEM and a JED-2300 EDS. The X-ray photoelectron spectroscopy (XPS) of the corrosion scale was examined using a Thermo Escalab 250Xi instrument.

## Results

### RCE test

For typical RCE devices, the transition from laminar to turbulent flow occurs when the Reynolds number exceeds 200. Because this transition occurs at a relatively low rotation rate, the RCE is considered an ideal tool for studying turbulent flow at a low velocity, which is a condition frequently observed in pipeline infrastructures. Moreover, higher turbulent velocities are easily achievable at higher rotation rates.

**Fig. 3:**
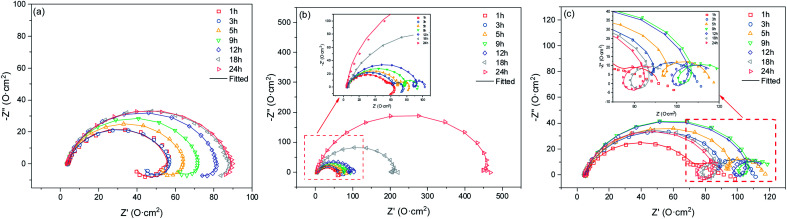
Nyquist diagrams (a) carbon steel, 2000 rpm; (b) 3Cr steel, 1000 rpm; (c) 3Cr steel, 2000 rpm.

The corrosion of 3Cr steel and carbon steel under different rotation rates was periodically investigated through EIS. EIS measurements can provide insight into the change of corrosion film characteristics and the corrosion process occurring at the interface. Fig. 3a presents the Nyquist diagrams of carbon steel obtained at 2000 rpm after immersion for 1, 3, 5, 9, 12, 18, and 24 h, with the characteristics of a capacitance loop and an inductive loop. The impedance increased slowly, which means the product film grows slowly, and the corrosion rate decreases slowly. A distinct inductive loop in the low frequency was always present. The low-frequency inductive loop is associated with the adsorption of the intermediate product on the substrate surface. It indicated that a corrosion film was barely formed on the carbon steel surface at a high flow rate. The adsorption and desorption of intermedia products on the steel surface were extremely high.

Fig. 3b presents the Nyquist diagrams of 3Cr steel obtained at 1000 rpm after immersion for 1, 3, 5, 9, 12, 18, and 24 h, with the characteristics of two typical capacitance loops and an inductive loop. The impedance gradually increased, which was attributed to corrosion scale growth on the substrate. Initially, the inductive loop expanded and then shrank. The inductive loop was closely related to the adsorption and desorption of intermediate products. In the initial stage, the corrosion film coverage was low, and the formation of intermediate products such as FeOH_ad_ and CrOH_ad_ increased with time. The adsorption and desorption of intermediate products on the steel surface became stronger. Subsequently, as corrosion proceeded, the coverage of the corrosion film increased, and the adsorption and desorption of intermediate products on the steel surface weakened.

Fig. 3c presents the Nyquist diagrams of 3Cr steel obtained at 2000 rpm after 1, 3, 5, 9, 12, 18, and 24 h of immersion, with the characteristics of two typical capacitance loops and an inductive loop. Initially, the impedance increased and then shrank, and the inductive loop gradually increased. In the initial stage, the corrosion products deposited on the steel surface and corrosion film coverage increased. When corrosion proceeded, the corrosion film was destroyed because of the high flow rate. Moreover, the formation of intermedia products increased with time, and the adsorption and desorption of intermedia products became stronger.

**Fig. 4:**
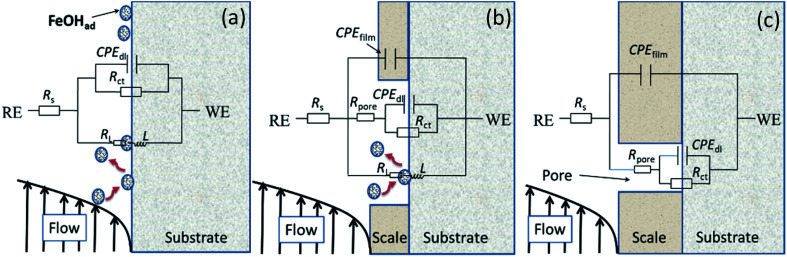
Equivalent circuits used for modeling the EIS results in Fig. 3.

**Fig. 5:**
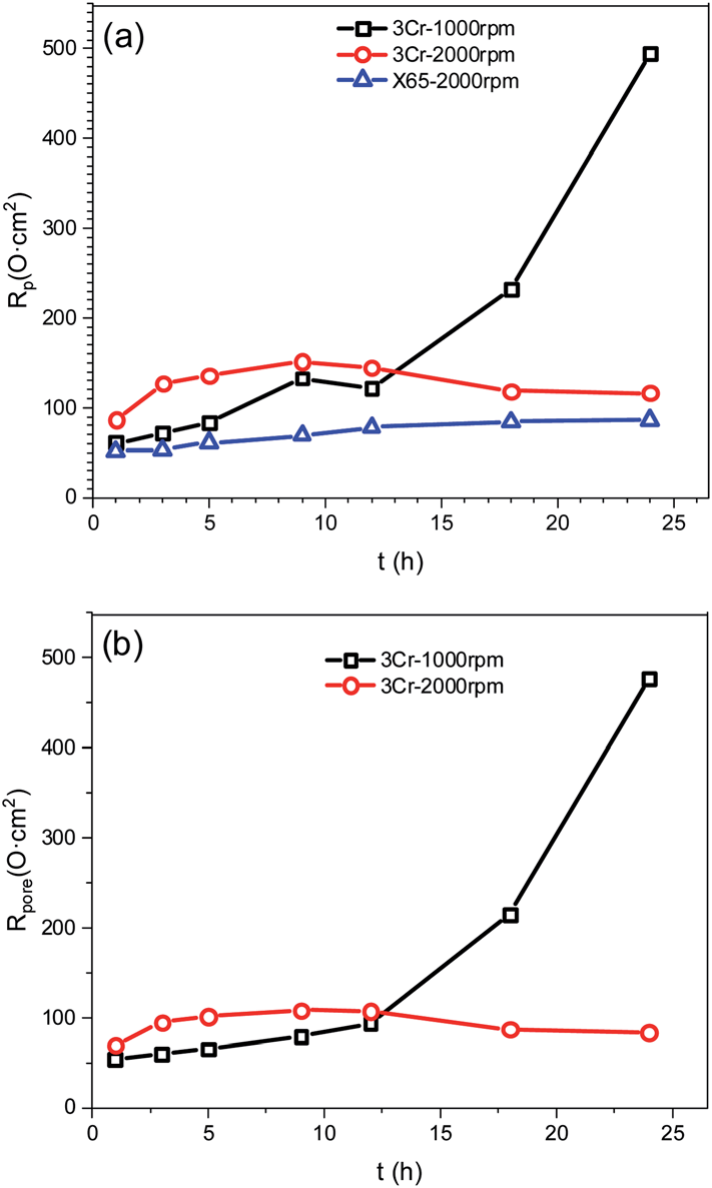
The *R*_pore_ value (a) and *R*_p_ value (b) obtained from the EIS results.

Fig. 4 illustrates equivalent circuits for EIS fitting. *R*_s_, CPE_film_, and *R*_pore_ denote the electrolyte solution resistance, constant phase element (CPE) used to fit the corrosion film capacitance, and resistance of the pores in the corrosion film, respectively. Electrochemical processes at the interface are represented by *R*_ct_ and CPE_dl_, which denote the charge transfer resistance and electric double-layer capacitance, respectively. *Y*_o_ and *n* were the parameters obtained using the constant phase element. *R*_L_ and *L* were the inductive resistance and inductance, respectively. Table 5 presents the results. The polarisation resistance (*R*_p_), that is, the sum of *R*_pore_ and *R*_ct_, was inversely proportional to the corrosion rate. For carbon steel or low Cr-bearing alloy steel, the corrosion medium can react with steel through pores present on a protective scale, and dissolved Fe^2+^ can exit the pores. Thus, protecting the corrosion scale is closely associated with the *R*_pore_. Fig. 5 presents *R*_pore_ and *R*_p_ obtained from Table 5.

**Table 5:** Values of the elements of the equivalent circuit in Fig. 4 to fit the impedance spectra of Fig. 3

Conditions	Immersion time, h	*R*_s_, Ω cm^2^	CPE_film_		*R*_pore_, Ω cm^2^	CPE_dl_		*R*_ct_, Ω cm^2^	*R*_L_, Ω cm^2^	*L*, H
			*Y*_1_, μΩ^−1^ cm^−2^ s^*n*^	*n*_1_		*Y*_2_, ×10^−4^ Ω^−1^ cm^−2^ s^*n*^	*n*_2_			
Carbon steel 2000 rpm	1	4.26				4.06	0.89	51.9	106.3	1432
	3	3.86				6.22	0.86	53.4	147.2	2073
	5	3.74				6.83	0.87	61.2	205.8	3031
	9	3.69				7.81	0.88	68.9	278.1	5733
	12	3.66				10.49	0.87	78.3	310.8	13 890
	18	3.66				16.09	0.85	84.4	268.1	27 630
	24	3.49				20.77	0.84	86.0	245.0	33 220
3Cr 1000 rpm	1	3.87	909	0.77	53.5	1463	1	7.21	62.6	9429
	3	4.31	703	0.81	59.5	3471	1	12.6	119	17 400
	5	4.71	616	0.82	65.1	4548	1	18.0	316	19 030
	9	4.61	702	0.78	79.2	1028	1	53.6	267	342.7
	12	4.77	622	0.80	93.4	3069	1	27.8	539	566.1
	18	5.81	353	0.83	215	6572	1	17.2	2089	3439
	24	3.17	244	0.86	477	6853	1	17.7	6299	13 560
3Cr 2000 rpm	1	5.06	785	0.79	69.8	1541	0.89	16.6		
	3	4.35	722	0.79	94.6	1208	1	32.4	605	278.4
	5	4.7	686	0.79	101	1479	1	35.2	604	305.3
	9	4.82	497	0.84	108	2244	0.83	43.4	596	488
	12	5	487	0.84	107	3517	0.89	37.8	586	497.3
	18	4.51	495	0.85	86.9	6721	0.98	31.6	436	388.9
	24	4.71	481	0.86	83.7	8928	1	32.6	392.9	417

The *R*_p_ value of carbon steel at 2000 rpm was considerably low and slowly increased with time, indicating that the corrosion film may form only negligibly on the steel surface. For 3Cr steel at 2000 rpm, *R*_p_ and *R*_pore_ increased first and then decreased, which was attributed to the formation and destruction of the corrosion film. The *R*_p_ of 3Cr steel was considerably higher than that of carbon steel, which revealed that the corrosion resistance of 3Cr steel at a high flow rate was substantially stronger than that of carbon steel. When the rotation speed decreased to 1000 rpm, *R*_p_ and *R*_pore_ sharply increased with time. In the initial stage (1–12 h), the value was lower than that at 2000 rpm, which indicated that the high flow rate could accelerate the formation of considerably more protective corrosion films on the 3Cr steel surface. After 12 h, the value at 1000 rpm rapidly exceeded that at 2000 rpm. This finding revealed that the rotation speed of 1000 rpm posed no threat to the corrosion film. At a high flow rate, 3Cr steel exhibited substantially better flow accelerated corrosion resistance than carbon steel did.

**Fig. 6:**
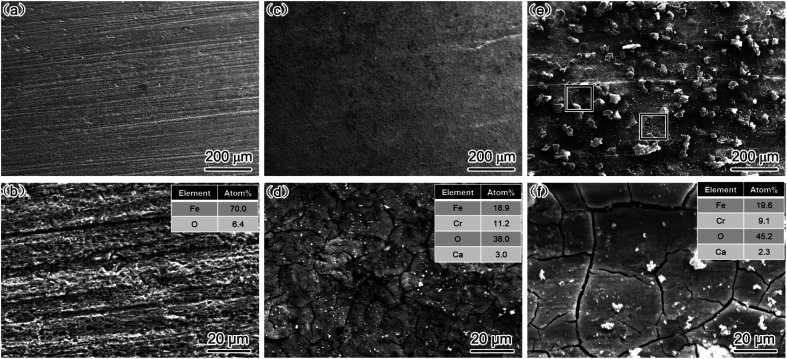
SEM-SE image and EDS analysis of corrosion scale (a and b) carbon steel, 2000 rpm; (c and d) 3Cr steel, 1000 rpm; (e and f) 3Cr steel, 2000 rpm.

The EIS results indicated that the addition of Cr improved the corrosion resistance of steel, mainly because Cr positively influences the deposition of a corrosion film on steel at a high flow rate. Therefore, to further confirm the aforementioned conjugation, SEM and EDS analyses were conducted, and the results are presented in Fig. 6.

For carbon steel, the SEM results revealed no prominent corrosion film on the steel surface. The EDS results indicated that the O and Fe concentrations were considerably lower and higher, respectively, in carbon steel than in 3Cr steel. This finding indicated that FeCO_3_ rarely deposited on the surface of steel under the condition of the high flow rate. The results are consistent with those presented in Fig. 3a.

For 3Cr steel, the surface was covered with the cracked corrosion film deposited at 1000 and 2000 rpm. The EDS results revealed that the corrosion film was Cr-enriched, indicating that the main components of the corrosion product film were Cr(OH)_3_ and FeCO_3_. The corrosion film can provide excellent protection for 3Cr steel at a high flow rate. In the produced film, the ratio of Cr/Fe at 1000 rpm was slightly higher than that at 2000 rpm, and the oxygen content was slightly higher at 2000 rpm than that at 1000 rpm. This may be because the corrosion rate at 2000 rpm was higher than that at 1000 rpm, which led to the generation of more Fe^2+^. Finally, the content of FeCO_3_ deposited on the corrosion film increased, and the corrosion film became thicker.

The corrosion film on the surface was complete without any damage at 1000 rpm, and at 2000 rpm, most of the corrosion film on the surface was complete, but some areas of the corrosion film were damaged.

This finding is consistent with the EIS results, that is, when the flow rate was 1000 rpm, the corrosion film on the surface grew steadily with time and exhibited excellent flow accelerated corrosion resistance, which was not damaged by fluids under this working condition.

When the rotation rate increased to 2000 rpm, the severe turbulence damaged the corrosion film. However, because most areas of the steel surface were covered with the complete corrosion film, the corrosion resistance of 3Cr steel remained considerably higher than that of carbon steel, which cannot form a continuous corrosion film.

### Flow loop test

The results of the RCE experiment indicated that adding Cr can enhance the corrosion resistance of steel at a high flow rate. Furthermore, to confirm the excellent flow accelerated corrosion resistance of low Cr-containing alloy steel at a high flow rate and its corrosion resistance mechanism, flow loop tests were conducted, which can simulate actual conditions in the wet gas pipeline.

**Fig. 7:**
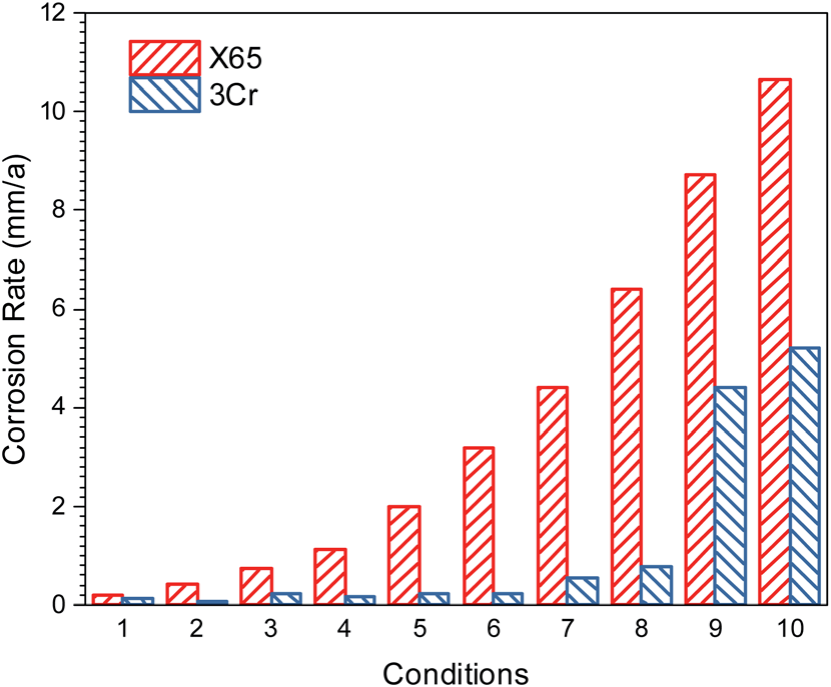
Corrosion rate of carbon steel and 3Cr steel measured under different conditions in Table 4.

Fig. 7 presents the corrosion rate of carbon steel and 3Cr steel measured under different conditions by conducting flow loop tests. The flow accelerated corrosion resistance of 3Cr steel was considerably better than that of carbon steel under wet gas conditions at a high flow rate.

**Fig. 8:**
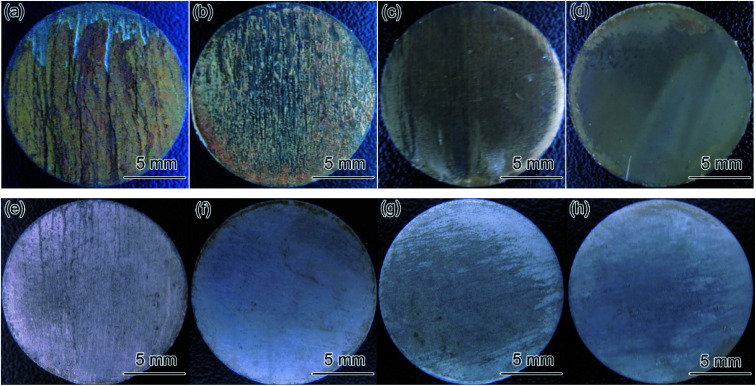
Macro morphologies of coupons with (a–d) and without (e–h) the corrosion film after flow loop tests under conditions 5 and 6 in Table 4: (a and e) carbon steel, 24 h; (b and f) carbon steel, 72 h; (c and g) 3Cr steel, 24 h; (d and h) 3Cr steel, 72 h.

Fig. 8 illustrates the macro morphologies of coupons with and without the corrosion film after 24 and 72 h flow loop tests under conditions 5 and 6 in Table 4. The corrosion film on carbon steel coupons exhibited obvious damage along the direction of fluid flow, resulting in some matrices being exposed, and the surface of 3Cr steel was covered with a complete corrosion product film. The result indicated that the corrosion film formed on the 3Cr steel surface exhibited considerably better flow accelerated corrosion resistance than that formed on the carbon steel surface did.

**Fig. 9:**
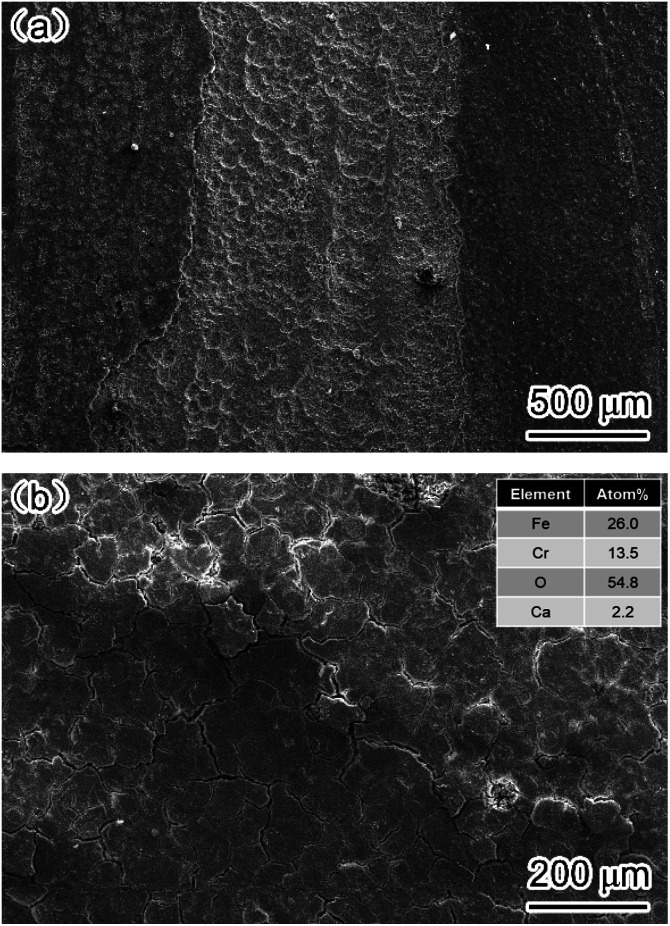
SEM-SE image of corrosion film on (a) carbon steel and (b) 3Cr steel after flow loop tests under condition 6 in Table 4.

**Fig. 10:**
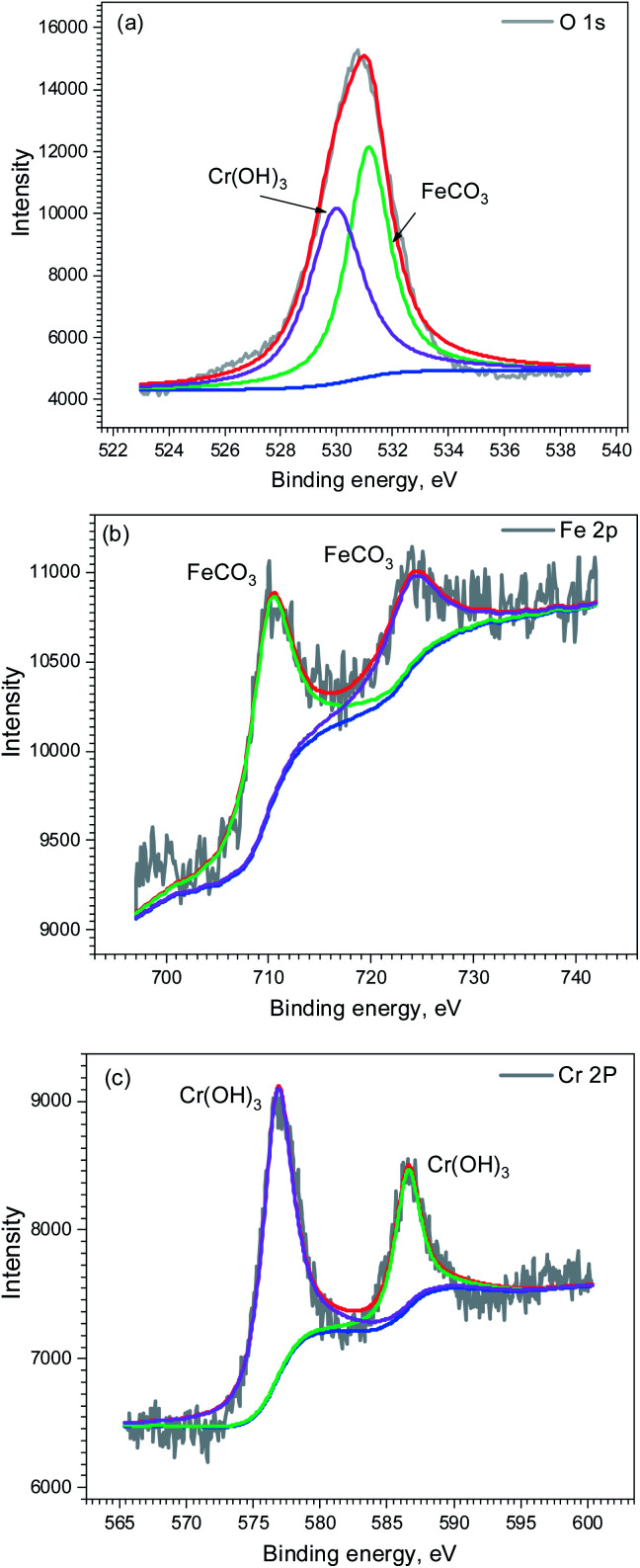
XPS analysis of corrosion scale formed on 3Cr steel surface after flow loop tests under condition 6 in Table 4: (a) O 1s; (b) Fe 2p_2/3_; (c) Cr 2p_2/3_.

Fig. 9 shows the surface morphology of corrosion film on the carbon steel and 3Cr steel surface after 72 h corrosion in the flow loop tests under condition 6. The corrosion film of carbon steel showed an obvious sign of being damaged by fluid erosion. However, the corrosion film of 3Cr steel was intact, and some cracks formed on the entire surface because of dehydration.^15,32^ The EDS results indicated that the corrosion film of 3Cr steel was rich in Cr. Furthermore, to determine the composition of the corrosion product film, the corrosion film was analyzed through XPS; Fig. 10 presents the results. The XPS results revealed that the corrosion film mainly comprises Cr(OH)_3_ and FeCO_3_.

**Fig. 11:**
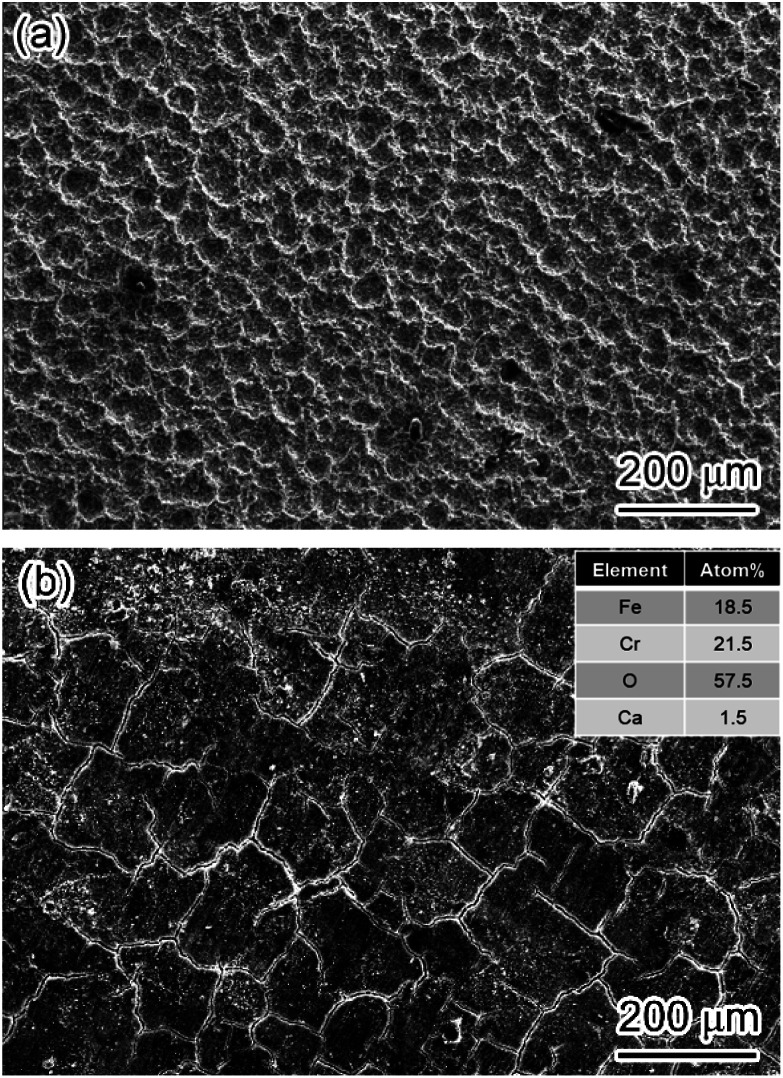
SEM-SE image of corrosion film on 3Cr steel after flow loop tests under condition 10 in Table 4. (a) Carbon steel, (b) 3Cr steel.

Furthermore, the corrosion films formed on X65 and 3Cr steel in the flow loop under the condition of the highest wall shear stress were analyzed through SEM and EDS. The carbon steel matrix exhibited a crater-like surface, and no prominent corrosion film was observed, which indicated that it is difficult for FeCO_3_ deposited onto the steel surface to form an intact corrosion film (Fig. 11a). However, a mixture of Cr(OH)_3_ and FeCO_3_ could still be deposited on the 3Cr steel surface and form an intact and protective corrosion film even under the conditions of 212 Pa wall shear stress (Fig. 11b), indicating that Cr(OH)_3_ can improve the shear resistance of corrosion films.

## Conclusions

The effect of chromium on the corrosion behaviour of low Cr-bearing steel under a high flow rate or wall shear stress was studied using RCE and flow loop tests. The main conclusions are as follows:

(1) The flow accelerated corrosion resistance of 3Cr steel is considerably better than that of carbon steel under a high flow rate, especially in a wet gas environment. Adding chromium to steel can effectively increase its flow accelerated corrosion resistance.

(2) It is difficult for pure FeCO_3_ deposited onto the carbon steel surface to form an intact corrosion film at a high flow rate. However, a mixture of Cr(OH)_3_ and FeCO_3_ could still be deposited on the 3Cr steel surface and form an intact and protective corrosion film even under the conditions of 212 Pa wall shear stress, indicating that Cr(OH)_3_ can improve the shear resistance of corrosion films.

## Conflicts of interest

There are no conflicts to declare.

## Supplementary Material

## References

[cit1] Zhang G. A., Cheng Y. F. (2010). Corros. Sci..

[cit2] Zhang G. A., Zeng L., Huang H. L., Guo X. P. (2013). Corros. Sci..

[cit3] Jiang X., Zheng Y. G., Ke W. (2005). Corros. Sci..

[cit4] Prethaler A., Mori G., Rosenberg E. (2015). BHM Berg- Hüttenmännische Monatsh..

[cit5] Dooley R. B., Chexal V. K. (2000). Int. J. Pressure Vessels Piping.

[cit6] Xu Y., Tan M. Y. (2019). Corros. Sci..

[cit7] Ajmal T. S., Arya S. B., Udupa K. R. (2019). Int. J. Pressure Vessels Piping.

[cit8] Abd A. A., Naji S. Z., Hashim A. S. (2019). Eng. Failure Anal..

[cit9] Kain V. (2014). Procedia Eng..

[cit10] Kim S., Kim T., Lee Y., Kim J. H. (2019). Corros. Sci..

[cit11] Zafar M. N., Rihan R., Al-Hadhrami L. (2015). Corros. Sci..

[cit12] Hassani S., Vu T. N., Rosli N. R., Esmaeely S. N., Choi Y. S., Young D., Nesic S. (2014). Int. J. Greenhouse Gas Control.

[cit13] Jiang S., Chai F., Su H., Yang C. F. (2017). Corros. Sci..

[cit14] Chai F., Jiang S., Yang C. (2016). J. Iron Steel Res. Int..

[cit15] Guo S., Xu L., Zhang L., Chang W., Lu M. (2016). Corros. Sci..

[cit16] Zhu J., Xu L., Lu M., Zhang L., Chang W., Hu L. (2015). Corros. Sci..

[cit17] Wang B., Xu L., Zhu J., Xiao H., Lu M. (2016). Corros. Sci..

[cit18] Yan W., Deng J., Li X., Dong X., Zhang C. (2012). Chin. Sci. Bull..

[cit19] Stack M. M., James J. S., Lu Q. (2004). Wear.

[cit20] De Motta R. A. , A Combined Experimental and Modelling Approach to Elucidate FeCO_3_ Scale Formation Kinetics, University of Leeds, 2016

[cit21] Nešić S. (2007). Corros. Sci..

[cit22] Yang Y., Brown B., Nešić S., Gennaro M. E., Molinas B. (2010). Corrosion.

[cit23] Utanohara Y., Murase M. (2019). Nucl. Eng. Des..

[cit24] Tomarov G. V., Shipkov A. A., Komissarova T. N. (2019). Therm. Eng..

[cit25] Bandla Y., Thamida S. K. (2019). J. Fail. Anal. Prev..

[cit26] El-Gammal M., Mazhar H., Cotton J. S., Shefski C., Pietralik J., Ching C. Y. (2010). Nucl. Eng. Des..

[cit27] Tao W., Shigeru S. (2018). Metals.

[cit28] Senatore E. V., Taleb W., Owen J. (2018). Wear.

[cit29] Mansoori H., Mirzaee R., Esmaeilzadeh F., Vojood A., Dowrani A. S. (2017). Eng. Failure Anal..

[cit30] Svenningsen G., Kvarekvål J. (2018). Corrosion.

[cit31] Jenkins A., Gilbert I. (2012). Corrosion.

[cit32] Chen C., Lu M., Sun D., Zhang Z., Chang W. (2005). Corrosion.

